# Antibiotic prescribing for adults with group A streptococcal bacteremia in a large healthcare system

**DOI:** 10.1017/ash.2023.449

**Published:** 2023-10-19

**Authors:** David A. Gillins, Mary Hutton, Whitney R. Buckel

**Affiliations:** 1 Department of Pharmacy, WVU Hospitals, Morgantown, WV, USA; 2 Department of Pharmacy, Intermountain Health Utah Valley Hospital, Provo, UT, USA; 3 Department of Pharmacy, Intermountain Health, Taylorsville, UT, USA

## Abstract

**Purpose:**

Limited data exist regarding treatment of invasive group A streptococcal (GAS) infections, including safety and efficacy of oral (PO) step-down therapy. We sought to describe current prescribing practices and clinical outcomes for patients with GAS bacteremia across a large health system, including a prespecified subset of patients who stepped down to PO antibiotics.

**Methods:**

This retrospective cohort study included adult patients with a positive blood culture for GAS between July 2018 and July 2021. Primary outcomes included frequency of PO step-down, total duration of therapy, duration of intravenous (IV) therapy prior to PO switch, and antimicrobial selection. Secondary outcomes included length of stay (LOS), mortality, adverse events, and clinical failure leading to readmission within 90 days.

**Results:**

In total, 280 patients met inclusion criteria. Of these, 46.7% were stepped down to PO antibiotics. Median total duration of therapy was 15 days. Median duration of IV therapy prior to PO switch was 5 days. The predominant definitive antibiotic choice was a beta-lactam. Median LOS was 5 days. Ninety-day mortality was 16.7%. One patient developed an occluded line and one developed *Clostridioides difficile*-associated diarrhea within 90 days. Ninety-day readmission due to clinical failure was 12.5%. Among cases of uncomplicated skin and soft tissue source, mortality (6.1% vs 2.4%) and readmission (15.2% vs 16.9%) were similar between definitive IV and PO groups.

**Conclusions:**

Group A streptococcal bacteremia is a severe infection with a high readmission and mortality rate. Use of PO step-down therapy was common with similar readmission and mortality rates compared with definitive IV therapy.

## Background

*Streptococcus pyogenes*, also known as Group A Streptococcus (GAS), is a gram-positive bacterium that causes clinical syndromes ranging from mild, localized infections, such as pharyngitis and impetigo, to invasive diseases, such as pneumonia, bacteremia, necrotizing fasciitis, and toxic shock syndrome.^1,2^ Invasive disease results in significant morbidity and mortality, with a case fatality rate of up to 48%.^1,3–9^ Invasive GAS infections have become increasingly prevalent in the United States over the past 2 decades, with 10,200 cases reported in 1998, in comparison with 25,160 cases in 2018.^10^ Despite the significant and growing burden of invasive disease, including bacteremia, no randomized controlled studies have been conducted to guide antimicrobial selection or duration of therapy.

Optimal antimicrobial selection for GAS bacteremia remains unclear. While macrolide resistance is now common in some communities due to frequent empiric use for respiratory infections, GAS isolates remain universally susceptible to penicillin.^11–13^ As such, the Centers for Disease Control and Prevention recommends penicillin or amoxicillin as the antibiotic of choice for GAS pharyngitis, with a first-generation cephalosporin among the suggested alternatives for patients with a penicillin allergy; however, no specific recommendations are provided for invasive disease.^13^ Despite susceptibility to penicillin, the most common IV antibiotic used for definitive therapy in recent retrospective streptococcal bacteremia studies was ceftriaxone.^14,15^ In contrast, definitive PO antibiotic selection varies across studies, with amoxicillin, amoxicillin/clavulanate, levofloxacin, and cefdinir each documented as most frequent.^14–18^

Similarly, there are no prospective data to inform optimal duration of therapy. A recent retrospective GAS bacteremia study out of tropical Australia found no difference in 90-day mortality or hospital readmission between patients treated with less than or equal to 10 days of antibiotic therapy and those treated with longer courses.^19^ Additionally, optimal timing of PO step-down is unclear. Nguyen and colleagues^19^ recently concluded no difference in 90-day mortality or hospital readmission between patients receiving less than or equal to 5 days of IV antibiotics prior to PO step-down and those receiving greater than 5 days. While these data suggest shorter durations of therapy and early PO step-down may be appropriate, uncertainty remains, particularly for GAS bacteremia, as studies have largely focused on other streptococcal species.

Gram-positive bloodstream infections (BSIs), including GAS, have historically been managed with IV antibiotics, which are associated with prolonged hospitalizations, increased healthcare costs, and line-related adverse effects.^20–23^ Due to these complications, there is increasing interest in using definitive PO therapy. In recent years, several studies have evaluated the use of step-down PO antibiotics for non-staphylococcal gram-positive BSIs, including uncomplicated streptococcal BSIs.^14–18^ Quinn and colleagues^16^ reported an 80% clinical success rate with PO step-down for non-staphylococcal gram-positive BSIs. Arensman and colleagues^17^ found PO step-down therapy with beta-lactams to be noninferior to fluoroquinolones for uncomplicated streptococcal BSI in terms of recurrence, readmission, mortality, and *Clostridioides difficile*-associated diarrhea (CDAD). Recent studies from Kang,^14^ Waked,^15^ and Ramos-Otero^15^ found that patients with uncomplicated streptococcal BSIs treated with PO step-down therapy had a significantly shorter LOS, while maintaining a similar rate of recurrence, readmission, adverse effects, and mortality.

While these studies suggest that PO step-down therapy is a safe and effective strategy, it is unclear whether this is appropriate for all streptococcal species. Notably, the most frequent organisms isolated on blood culture in these studies were *Streptococcus pneumoniae* (19.3%–34.1%), a member of the viridans group Streptococcus family (20.0%–41.8%), or Group B Streptococcus (15.5%–22.7%), while GAS was less common, representing just 6.8% to 20.4% of cases.^14–18^ There are no data directly comparing the safety and efficacy of PO step-down therapy to definitive IV antibiotics specifically in the setting of GAS bacteremia. The primary purpose of this study was to describe the current prescribing practices and clinical outcomes for GAS bacteremia across a large, integrated healthcare system, including a prespecified subset of patients who were stepped down to PO therapy.

## Methods

This is an IRB-approved, retrospective, observational, multi-site, cohort study of patients admitted to an acute care hospital with a positive blood culture for GAS from July 1, 2018, through July 31, 2021. During the study period, the health system consisted of 23 hospitals, including one large medical center, 4 large community hospitals, and 18 small (less than 200 beds) community or critical access hospitals. Antimicrobial stewardship is conducted in the health system through prospective audit and feedback. A third-party software, VigiLanz®, is utilized for culture surveillance. Front-line and infectious diseases pharmacists review all positive blood cultures daily and Monday through Friday, respectively.

Electronic medical records were reviewed to identify patients with an initial positive blood culture for GAS during the study period. Patients less than 18 years of age and those transferred to a facility outside of our health system were excluded. A secondary analysis was conducted to provide a comparison between definitive IV and PO step-down therapy in patients with uncomplicated bacteremia of skin and soft tissue (SSTI) source. We defined definitive therapy as the antibiotic regimen selected to complete therapy, pursuant to culture results. For this secondary analysis, the following patients were also excluded: patients that underwent a surgical source control procedure (defined as drainage of abscess or fluid collection and/or debridement of infected tissue), patients that received less than 7 days of antimicrobial therapy, and patients with a concurrent infection requiring a greater than 2-week duration of antimicrobial therapy. Demographic characteristics and clinical data for patients meeting inclusion criteria were collected from electronic medical records and Pitt Bacteremia and Charlson Comorbidity Index scores were calculated.

Prescribing practices of interest were infectious diseases consultation, antibiotic selection, total duration of antibiotic therapy, frequency of PO step-down therapy, and duration of IV antibiotic therapy prior to PO switch. Clinical outcomes included LOS, 90-day mortality, adverse events, and clinical failure leading to readmission within 90 days. We defined clinical failure as unresolved or worsening infection despite active antibiotic therapy. A patient was determined to have an unresolved or worsening infection when persistent or evolving infectious symptoms were described by the physician in the electronic medical record.

## Results

### Patient characteristics

A total of 316 patients had an initial positive blood culture for GAS during the study period. Among these, 36 (11.4%) patients were excluded, 24 (7.6%) of which were under the age of 18 and 12 (3.8%) were transferred to a facility outside of our health system, leaving 280 patients meeting the study criteria. Of the 280 patients, 206 (73.6%) patients had an SSTI source of infection. Of note, 55 (19.6%) patients endorsed current injection drug use. Additional patient characteristics are summarized in Table 1.

**Table 1.** Baseline characteristics

Characteristic	Total (*N* = 280)
Age (years) [median (IQR)]	61 (44–74)
Female sex [*n* (%)]	118 (42.1)
White race [*n* (%)]	261 (93.2)
Pitt bacteremia score [median (IQR)]	2 (0–3)
Charlson Comorbidity Index [median (IQR)]	4 (1–6)
Source of infection [*n* (%)]	–
SSTI	206 (73.6)
Respiratory	29 (10.4)
Oropharyngeal	11 (3.9)
Other^a^	10 (3.6)
Unknown	24 (8.6)
PWID [*n* (%)]	55 (19.6)
ID consultation [*n* (%)]	131 (46.8)

Note. ID, infectious diseases; IQR, interquartile range; PWID, person who injects drugs; SSTI, skin and soft tissue infection.

a Other infrequent sources of infection included endocarditis, endometriosis, spinal hardware infection, septic arthritis, and urinary.

### Prescribing practices

Vancomycin and ceftriaxone were the most frequent initial antibiotic selections, given to 177 (63.2%) and 149 (53.2%) patients, respectively. Beta-lactams were predominantly selected for definitive treatment, with 209 (74.6%) patients prescribed a beta-lactam and 38 (13.6%) patients prescribed a non-beta-lactam antibiotic. Ten of the patients prescribed a non-beta-lactam antibiotic had a documented beta-lactam allergy. The median total duration of therapy was 15 days (interquartile range [IQR] 12–19 days). Overall, 98 (35.0%) patients were treated with definitive IV antibiotics, 131 (46.7%) were treated with definitive PO antibiotics, 6 (2.1%) were treated with a combination of IV and PO definitive antibiotics, and 45 (16.1%) did not receive definitive antibiotics due to in-hospital mortality, discharge on hospice without antibiotics, or discharge against medical advice. In those who were treated with PO step-down therapy, the median duration of IV antibiotics prior to PO switch was 5 days (IQR 3–7 days). Additional outcomes are described in Table 2. For a complete breakdown of definitive antimicrobial selection, please see Table 3.

**Table 2.** Prescribing practices and clinical outcomes

Variable	Total (*N* = 280)
Initial antibiotic selection [*n* (%)]	–
Vancomycin	177 (63.2)
Ceftriaxone	149 (53.2)
Piperacillin/tazobactam	59 (21.1)
Cefepime	28 (10.0)
Clindamycin	28 (10.0)
Definitive antibiotic selection [*n* (%)]	–
Definitive IV	98 (35.0)
Beta-lactam	86 (87.8)
Non-beta-lactam	10 (10.2)
BL/NBL combination	2 (2.0)
Definitive PO	131 (46.7)
Beta-lactam	111 (84.7)
Non-beta-lactam	15 (11.5)
BL/NBL combination	5 (3.8)
Definitive IV/PO combination	6 (2.1)
No definitive antibiotic therapy	45 (16.1)
In-hospital mortality	35 (12.5)
Discharged on hospice without antibiotics	6 (2.1)
Discharged AMA without antibiotics	4 (1.4)
Total duration of therapy (days) [median (IQR)]	15 (12–19)
Duration of IV antibiotics prior to PO switch (days) [median (IQR)]	5 (3–7)
Length of stay (days) [median (IQR)]	5 (3–8)
90-day mortality [*n* (%)]	47 (16.7)
Adverse events [*n* (%)]	–
*Clostridioides difficile* infection	1 (0.4)
Occluded PICC line	1 (0.4)
Ninety-day readmission due to clinical failure [*n* (%)]	35 (12.5)

Note. AMA, against medical advice; BL, beta-lactam; IQR, interquartile range; IV, intravenous; NBL, non-beta-lactam; PICC, peripherally inserted central catheter; PO, oral.

**Table 3.** Definitive antibiotics

Variable	Total (*N* = 280)
Definitive antibiotic selection [*n* (%)]^a^	–
Beta-lactam	209 (74.6)
Amoxicillin^b^	69 (24.6)
Amoxicillin/clavulanate^c^	11 (3.9)
Ampicillin	1 (0.4)
Ampicillin/sulbactam	2 (0.7)
Cefazolin^d^	22 (7.9)
Cefdinir	2 (0.7)
Cefepime	2 (0.7)
Ceftriaxone^e^	53 (18.9)
Cefuroxime	3 (1.1)
Cephalexin^f^	29 (10.4)
Ertapenem	1 (0.4)
Penicillin G^g^	10 (3.6)
Penicillin VK	3 (1.1)
Piperacillin/tazobactam	2 (0.7)
Non-beta-lactam	38 (13.6)
Ciprofloxacin	1 (0.4)
Clindamycin	3 (1.1)
Daptomycin	1 (0.4)
Doxycycline	4 (1.4)
Levofloxacin	4 (1.4)
Linezolid	7 (2.5)
Metronidazole	3 (1.1)
Trimethoprim/sulfamethoxazole	5 (1.8)
Vancomycin	12 (4.3)
No definitive antibiotic therapy	45 (16.1)

a The sum of definitive antibiotics and patients that received no definitive antibiotic therapy here is 292, as 12 patients were prescribed both a beta-lactam and a non-beta-lactam.

b Most frequent amoxicillin dose: 1 g PO TID (65.2%).

c Most frequent amoxicillin/clavulanate dose: 875–125 mg PO BID (63.6%).

d Most frequent cefazolin dose: 2 g IV Q8h (54.5%).

e Most frequent ceftriaxone dose: 2 g IV Q24h (94.3%).

f Most frequent cephalexin dose: 500 mg PO QID (55.2%).

g Most frequent penicillin G dose: 24 million units IV Q24h via continuous infusion (90.0%).

### Clinical outcomes

The median LOS was 5 days (IQR 3–7 days). The 90-day, all-cause mortality rate was 16.7%, with an in-hospital mortality rate of 12.5%. There was a low rate of documented adverse effects, with 1 documented case of CDAD and 1 documented occluded peripherally inserted central catheter. The 90-day readmission rate due to clinical failure was 12.5%. These outcomes are summarized in Table 2.

### Subgroup analysis

A total of 116 patients met inclusion criteria for the secondary analysis, with 33 patients in the definitive IV group and 83 patients in the definitive PO group. Patients in the definitive IV group had a higher median Pitt Bacteremia score (2 vs 1) and Charlson Comorbidity index (5 vs 3). In addition, a higher proportion of patients in the definitive IV group were females. Median patient age was similar between groups. The median total duration of therapy was 15 days (IQR 14–18 days) for the IV group and 14 days (IQR 12–17 days) for the PO group. The median LOS was 6 days (IQR 4–9.5 days) for the IV group and 5 days (IQR 3–6 days) for the PO group. The 90-day all-cause mortality rate was 6.1% for the IV group and 2.4% for the PO group. Again, there was a low documented rate of adverse effects, with one documented case of CDAD. These outcomes are summarized in Table 4.

**Table 4.** Skin and soft tissue source definitive IV vs. PO comparison

Variable	IV (*N* = 33)	PO (*N* = 83)
Age (years) [median (IQR)]	61 (44.5–72.5)	58 (45–75)
Female sex [*n* (%)]	17 (51.5)	34 (41.0)
White race [*n* (%)]	29 (87.9)	80 (96.4)
Pitt bacteremia score [median (IQR)]	2 (1–3)	1 (0–3)
Charlson Comorbidity Index [median (IQR)]	5 (2.5–7)	3 (1–6)
Total duration of therapy (days) [median (IQR)]	15 (14–18)	14 (12–17)
Length of stay (days) [median (IQR)]	6 (4–9.5)	5 (3–6)
Ninety-day mortality [*n* (%)]	2 (6.1)	2 (2.4)
In-hospital mortality [*n* (%)]	1 (3.0)	0
Adverse events [*n* (%)]	–	–
*Clostridioides difficile* infection	0	1 (1.2)
Occluded PICC line	0	–
Ninety-day readmission due to clinical failure [*n* (%)]	5 (15.2)	14 (16.9)

Note. IQR, interquartile range; IV, intravenous; PICC, peripherally inserted central catheter; PO, oral.

## Discussion

This study provides a valuable description of the GAS bacteremia patient population and clinical outcomes within a large, integrated healthcare system with an established antimicrobial stewardship program. With a relatively large patient population, this study provides additional insight specifically into the management and outcomes associated with GAS bacteremia, including a large cohort who received oral step-down therapy.

To our knowledge, this is the first study conducted in the United States describing prescribing practices and clinical outcomes for a study population comprised entirely of patients with GAS bacteremia. Previous reports that included patients with GAS bacteremia have also included either all non-staphylococcal gram-positive organisms or all streptococcal species.^14–18^ A recent study out of tropical Australia by Nguyen and colleagues^19^ was exclusive to patients with GAS bacteremia, but primarily focused on duration of therapy. Patients in the current report were of a similar age (median 61 yr vs 60 yr) with a comparable mortality risk (median Charlson Comorbidity index 4 vs 4) to patients included in the Nguyen study.^19^ Similarly, the primary source of infection in this study and the Nguyen study^19^ was SSTI (73.6% vs 71.3%). Notably, we observed a high rate of injection drug use (19.6%), which is consistent with available literature on invasive GAS disease, with prevalence of injection drug use reported as high as 28%, with a range of 5.5%–28% in previous reports.^1,3,14,18,24,25^

A lower rate of ID consultation (46.8%) was observed in this study than has been reported in previous reports (50.0%–85.2%).^16,17,19^ However, as two out of three prior studies reporting on the rate of ID consultation were limited to patients who survived until discharge and were stepped down to PO antibiotics, the lower rate of ID consultation in this report is not unexpected.^16,17^ The most common definitive IV antibiotic was ceftriaxone, which was also most common among previous streptococcal bacteremia studies.^14,15^ Ceftriaxone, an extended-spectrum cephalosporin, is unnecessarily broad for the treatment of GAS, which remains universally susceptible to penicillin.^11–13^ Despite this, only 13 of 280 patients (4.6%) in the current report were treated with IV or PO penicillin. The most common definitive PO antibiotic was amoxicillin, most frequently at a dose of 1 g PO 3 times daily. Amoxicillin, amoxicillin/clavulanate, levofloxacin, and cefdinir have been listed as the most common definitive PO antibiotics in previous non-staphylococcal gram-positive and streptococcal bacteremia studies.^14–18^ While the use of amoxicillin/clavulanate has generally been more common than amoxicillin in prior studies, the beta-lactamase inhibitor clavulanate is unnecessary for streptococcal infections. Similarly, fluoroquinolones are unnecessarily broad and carry significant collateral damage risks, while cefdinir is excessively broad and has very limited oral bioavailability (16%–21%).^26^ Utilization of narrow-spectrum beta-lactam therapy, namely amoxicillin (or alternatively, penicillin or cephalexin), may be a potential future target for stewardship programs in the treatment of streptococcal bacteremia, including GAS. Of note, amoxicillin may be more convenient in the outpatient setting, as it is dosed three times daily, whereas penicillin and cephalexin require four times daily dosing.

We observed a PO antibiotic switch rate of 46.7%, which is consistent with previous streptococcal bacteremia studies, which have reported PO antibiotic switch rates ranging from 40.2 to 52.0%.^14,15,18^ The median duration of IV antibiotics prior to PO switch in the current report was 5 days, which is comparable with previous non-staphylococcal gram-positive bacteremia studies and streptococcal bacteremia studies, which observed median durations of 4 to 5 days.^14,16–18^ Notably, Nguyen and colleagues^19^ recently found that patients with GAS bacteremia treated with less than or equal to 5 days of IV antibiotics prior to PO switch had similar outcomes to those that received greater than 5 days. Similarly, Ramos-Otero and colleagues^18^ observed similar outcomes for patients with streptococcal bacteremia treated with less than or equal to 3 days of IV antibiotics prior to PO switch had similar outcomes to those that received greater than 3 days. The findings of these recent retrospective studies suggest that it may be appropriate to shorten the duration of IV antibiotics prior to PO switch to less than or equal to 5 or even less than or equal to 3 days in some patients. This points to the need for a prospective study comparing clinical outcomes for patients receiving shorter and longer durations of IV therapy prior to PO switch.

The observed rate of hospital readmission due to clinical failure (12.5%) was relatively low. Quinn and colleagues^16^ previously reported a readmission due to clinical failure rate of 18.4% among patients with non-staphylococcal gram-positive bacteremia. Readmission primarily resulted from recurrent SSTIs in the current report, which is described in the existing literature for beta-hemolytic streptococcus infections.^27–29^ There were no observed cases of recurrent GAS bacteremia, which is described as uncommon in the literature.^14,16,17,28^ The observed 90-day all-cause mortality rate (16.7%) was consistent with existing literature, with prior studies reporting rates ranging from 5.6% to 48%.^3–9,19^ While Nguyen and colleagues^19^ observed a lower 90-day all-cause mortality rate (5.6%), most deaths in that study and the current report occurred in-hospital, accounting for 81.3% and 74.5% of deaths, respectively. Clinical outcomes observed in the IV versus PO comparison are encouraging and consistent with existing literature, with the two cohorts having similar mortality and readmission rates, but with the PO cohort having a shorter median LOS. This, along with most deaths occurring in-hospital, may suggest that most patients who are deemed medically ready for discharge can be effectively treated with PO step-down therapy. The observed rate of adverse effects was low for both cohorts, which is consistent with existing literature. Notably, the PO cohort had a lower Pitt bacteremia score and Charlson Comorbidity Index, suggesting that the IV cohort had a higher severity of illness and mortality risk.

Our study had several limitations. As a retrospective study, data collection was limited to adequate documentation in the medical record. We were unable to assess medication adherence after discharge, and any adverse effects or clinical failures identified at outside facilities would have gone undetected. However, as an integrated health system, we were able to identify patients who presented at any facility across our health system, increasing our overall capture rate. A significant portion of patients met exclusion criteria for the subgroup comparison of definitive IV versus definitive PO antibiotic therapy. We therefore did not conduct statistical analyses to compare definitive groups, as this would have been limited by insufficient statistical power and may have been misleading.

In conclusion, GAS bacteremia is a severe infection with a high mortality rate, with most deaths in this study occurring during the index admission. We did not observe any cases of recurrent bloodstream infection but did observe recurrent SSTIs. Transition to PO therapy was common after a median duration of 5 days of IV therapy. Among patients with uncomplicated GAS bacteremia of an SSTI source, similar readmission and mortality rates were observed between the definitive IV and PO groups. The findings of this study point to the need for further investigation in a comparative prospective trial comparing definitive IV therapy to PO therapy.
